# Task Offloading Decision-Making Algorithm for Vehicular Edge Computing: A Deep-Reinforcement-Learning-Based Approach

**DOI:** 10.3390/s23177595

**Published:** 2023-09-01

**Authors:** Wei Shi, Long Chen, Xia Zhu

**Affiliations:** 1 School of Computer Science and Engineering, Southeast University, Nanjing 211189, China; 220214782@seu.edu.cn; 2 The Key Laboratory of Computer Network and Information Integration (Southeast University), Ministry of Education, Nanjing 211189, China

**Keywords:** computation offloading, vehicular edge computing, deep reinforcement learning

## Abstract

Efficient task offloading decision is a crucial technology in vehicular edge computing, which aims to fulfill the computational performance demands of complex vehicular tasks with respect to delay and energy consumption while minimizing network resource competition and consumption. Conventional distributed task offloading decisions rely solely on the local state of the vehicle, failing to optimize the utilization of the server’s resources to its fullest potential. In addition, the mobility aspect of vehicles is often neglected in these decisions. In this paper, a cloud-edge-vehicle three-tier vehicular edge computing (VEC) system is proposed, where vehicles partially offload their computing tasks to edge or cloud servers while keeping the remaining tasks local to the vehicle terminals. Under the restrictions of vehicle mobility and discrete variables, task scheduling and task offloading proportion are jointly optimized with the objective of minimizing the total system cost. Considering the non-convexity, high-dimensional complex state and continuous action space requirements of the optimization problem, we propose a task offloading decision-making algorithm based on deep deterministic policy gradient (TODM_DDPG). TODM_DDPG algorithm adopts the actor–critic framework in which the actor network outputs floating point numbers to represent deterministic policy, while the critic network evaluates the action output by the actor network, and adjusts the network evaluation policy according to the rewards with the environment to maximize the long-term reward. To explore the algorithm performance, this conduct parameter setting experiments to correct the algorithm core hyper-parameters and select the optimal combination of parameters. In addition, in order to verify algorithm performance, we also carry out a series of comparative experiments with baseline algorithms. The results demonstrate that in terms of reducing system costs, the proposed algorithm outperforms the compared baseline algorithm, such as the deep Q network (DQN) and the actor–critic (AC), and the performance is improved by about 13% on average.

## 1. Introduction

As 5G technology and artificial intelligence are rapidly developing, autonomous driving has become a hot topic in both academic and engineering fields, particularly in intelligent transportation [1]. By leveraging advanced environmental perception and precise vehicle control, autonomous driving technology offers convenience and improves driving efficiency [2]. However, the increasing volume of in-vehicle data poses challenges for intelligent transportation systems [3]. The Internet of Vehicles, as an integral part of the Internet of Things, plays a crucial role in Intelligent Transport Systems, integrating vehicles, communication networks, and cloud computing [4,5]. Through Vehicle Ad Hoc Networks and various communication modes like Vehicle-to-Everything, IoV aims to enhance traffic safety, reduce congestion, and improve the overall user experience [6,7,8,9]. However, the proliferation of intelligent vehicles results in a surge of data and complex computational tasks [10], posing challenges such as limited network bandwidth, high latency, and security concerns [11]. To address these challenges, mobile edge computing moves computing and storage functions to the network edge as the complement of cloud computing, and becomes a promising solution [12,13,14]. This approach, suitable for vehicular networking environments requiring latency and reliability needs, alleviates IoV limitations. Vehicular edge computing combines mobile edge computing and the Internet of Vehicles, enabling distributed edge servers to facilitate computing offloading and interaction between vehicle users and roadside units via wireless access networks [15,16]. While mobile edge computing effectively addresses the limited resources and real-time constraints of the Internet of Vehicles, it still faces challenges such as restricted computing, storage, and bandwidth resources compared to cloud servers [17,18]. Overloading edge servers leads to increased service delay and reduced user experience [19]. Moreover, in vehicular edge computing where vehicles are the main research object, their high-speed mobility results in dynamic changes in communication links within the computing offloading environment [20]. Therefore, some service requests from vehicle users still require execution in the cloud or locally to optimize the utilization of system resources. Consequently, it remains a challenge to maintain the efficient operation of a vehicular edge computing system by designing intelligent resource management and scheduling schemes that can dynamically adapt to the complex vehicular edge computing environment while considering the dynamic nature of system status and resource constraints.

In the face of the challenge, traditional optimization techniques such as convex or non-convex optimization are insufficient to address decision-making problems in the vehicular edge computing environment. We introduce Deep Reinforcement Learning as our solution. Deep Reinforcement Learning is a field within artificial intelligence that enables intelligent agents to learn optimal actions through iterative interactions with the environment, allowing them effective adaptation to dynamic environments [21,22]. Inspired by the aforementioned issues, we design a VEC system model about “cloud-edge-vehicle” three-tier architecture, which integrates system resources, vehicle mobility, and task offloading to develop a comprehensive offloading strategy that considers both user experience and system energy consumption. This strategy effectively reduces system costs by making decisions on the task offload destinations of vehicles and reasonably allocating the proportion of task offloading. We formulate the task offloading decision process in vehicular edge computing environments as a Markov decision process. To optimize system costs, we propose a novel deep reinforcement learning algorithm that considers dynamic communication conditions. This algorithm effectively allocates the task offloading proportion, resulting in improved efficiency and reduced system costs.

The main contributions of this article are as follows:

- This paper proposes a “cloud-edge-vehicle” collaborative vehicular edge computing system model aimed at minimizing system costs by considering both system energy consumption and user experience. Taking into account the dynamic state of the system, we jointly optimize vehicle scheduling, task offloading and resource allocation. The optimization process is formulated as a Markov decision process and corresponding parameters are set.
- Taking into account the complexity of vehicular edge computing environment and continuous action space requirement of computation offloading decisions, this paper proposes a task offloading decision-making algorithm based on deep deterministic policy gradient (TODM_DDPG). The algorithm employs DNN networks to approximate the policy and Q-functions, thereby preventing dimensional explosion and obtaining the optimal policy by jointly addressing task offloading and resource allocation.
- This paper also provides a detailed training and validation process of the TODM_DDPG algorithm and introduces a state normalization mechanism to pre-process the system state.

The remaining structure of this paper is as follows: Section 2 examines previous related work. The system model and problem descriptions are introduced in Section 3. The background information of deep reinforcement learning and the suggested TODM_DDPG algorithm are introduced in Section 4. Section 5 investigates the experiment results. The paper is finally summarized in Section 6.

## 2. Related Work

As autonomous driving technology continues to evolve and intelligent transportation systems (ITS) mature, the scale of vehicular data for new applications such as vision-based object detection, path planning, and in-vehicle entertainment is growing explosively [23]. To address the diverse service needs of vehicular users, it is urgent to design effective computation offloading schemes for vehicular tasks. Due to its ability to meet the time-sensitive requirements of ITS application services, in the subject of intelligent transportation, the combination of mobile edge computing with the Internet of Vehicles has drawn considerable attention [15]. Computation offloading, as a core technology of mobile edge computing, encompasses several key elements, including the offloading target and destination, offloading mode, offloading function, and evaluation criteria for decision-making. These aspects collectively contribute to the overall process of computation offloading, enabling efficient resource utilization and improved system performance.

From the perspective of evaluation criteria for offloading decisions, most offloading decisions aim to optimize delay, energy consumption, system resources, combination of delay and energy consumption and maximize the quality of user experience or minimize the customized system cost. Luo et al. [24] aimed at minimizing offloading delay, proposed a multi-objective particle swarm optimization method using game theory analysis, which comprehensively considered communication, offloading decisions, and the allocation of computing resources. Simulation results show that this strategy is effective and feasible to solve the Pareto optimal solution. Based on the combination of a genetic algorithm and heuristic rules, Sun et al. [25] introduced a hybrid algorithm. Their approach aims to minimze delay and resource consumption by focusing on determining the execution location and the order of tasks. By leveraging the genetic algorithm and heuristic rules, their algorithm can effectively optimize task execution, resulting in reduced delays and efficient utilization of system resources. By introducing methods such as congestion degree and gravity reference point, literature [26] suggested an improved multi-objective whale optimization algorithm. A distributed computation offloading problem was presented in the senario of mobile device users by Chen et al. [27]. They presented a distributed technique that achieves the Nash equilibrium and formulated the problem as a game of multi-user computation offloading. Huang et al. [28] presented a cloud-edge server collaborative computation offloading method that leverages service orchestration. Their approach considers the delay and energy consumption and incorporates differentiated offloading decisions based on varying needs and delay sensitivities. By maximizing the distribution of processing tasks between cloud and edge servers, this technique successfully satisfies the demands of minimal latency and high dependability. In an effort to balance energy consumption and computation offloading delay, Cui et al. [29] suggested an enhancement to the NSGA-II method to discover the better solution to this constrained multi-objective optimization problem.

However, in the vehicular edge computing environment, the research object of computation offloading is the vehicle, while the mobility of the vehicle can lead to dynamic changes in the network topology. Traditional convex optimization or heuristic algorithms are not suitable for dynamic vehicular networks with vehicles, resources, and channel states. The majority of the aforementioned works, however, simply take into account the optimal or nearly optimal solution of the current system snapshot by offloading strategy, without considering the long-term impact of the current strategy on the environment. The decision-making process of computation offloading can be abstracted as a Markov decision process in the mobile edge computing context with dynamically changing network topology and computing resources [30]. With the help of repetitive interactions with the environment, reinforcement learning, a subfield of artificial intelligence, enables intelligent agents to learn the optimal action to maximize cumulative rewards. Reinforcement learning effectively addresses the Markov decision problem, permitting agents to learn through continuous trial and error [31]. A synchronous joint Q-learning (Sync-FQL) approach was introduced by Xiong et al. [32] that considers the probability of offloading failures in the vehicular edge computing environment while minimizing computation and communication costs. The algorithm optimizes the model to optimally utilize available resources by learning the Q-values from different parts. However, the performance of Q-learning algorithms in high-dimensional observation spaces is constrained because of the Q-table size constraint [33]. To overcome the shortcomings of conventional reinforcement learning algorithms in handling high-dimensional state and action spaces, the combination of deep learning with RL offers a potent strategy [21]. By leveraging the representation learning capabilities of deep neural networks, this combined approach enables effective learning and decision-making in complex environments. To improve computation offloading efficiency in vehicular networks, Zhao et al. [34] introduced an AHP-DQN-based computation offloading algorithm. This algorithm utilizes the Analytic Hierarchy Process (AHP) to appropriately allocate vehicle tasks and determines offloading decisions based on real-time channel gains. It addresses challenges such as limited terminal storage capacity and diverse network services in the process of computation offloading. In order to minimize user costs, Wang et al. [35] adopted the Double Deep Q-learning (DDQN) algorithm, treating the computational capacity of the mobile edge computing servers as the system state and improving resource utilization by learning offloading policies. For resource allocation and computation offloading, Khayyat et al. [36] introduced a DQN-based distributed learning algorithm. They approached the problem as a binary optimization challenge with the goal of minimizing the system’s overall time and energy costs. In order to meet the low latency requirements of vehicle-to-vehicle communication links and improve the throughput of vehicle-to-infrastructure links, Fu et al. [37] proposed the Double deep Q-learning (DDQN) algorithm, which effectively achieves the intelligent allocation of resources. While DQN-based algorithms effectively address dynamic computation offloading challenges, discretization methods used for continuous action spaces, such as resource allocation, can result in dimensionality explosion [38]. Regarding the computation offloading decision-making in continuous action spaces, research has been conducted recently. A mobile fog technique based on the DDPG algorithm, put forth by Chen et al. [39], effectively solves the state space explosion problem. Lu et al. [40] proposed an improved algorithm based on DDPG to solve the joint optimization problem of service delay, energy consumption and task success rate in the mobile edge computing environment, and effectively improved the quality of user experience.

## 3. System Model and Problem Description

**Figure 1:**
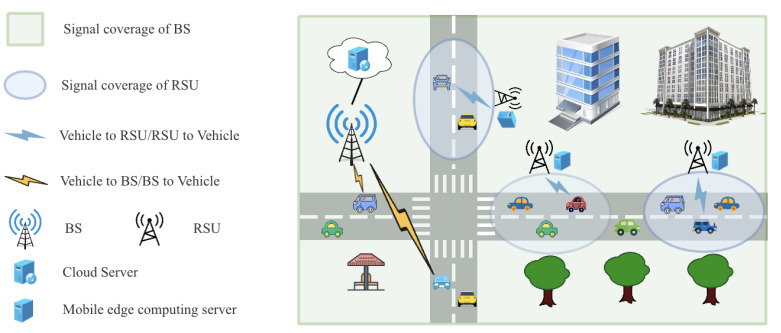
Architecture of vehicular edge computing network.

As illustrated in Figure 1, the suggested system adopts a three-tier, region-based architecture for vehicular edge computing network. They are vehicle layer, edge layer and cloud layer. The vehicle layer comprises user vehicles on the road that are equipped with limited computing resources. Each vehicle can communicate with base stations and roadside units through 5G/LTE technology [41] and a dedicated wireless interface (IEEE 802.11p). The edge layer comprises roadside nodes that are deployed in different regions of the map. These roadside nodes include roadside units with limited signal coverage range and mobile edge computing servers connected to roadside units with computational and storage resources, and the signal ranges between them do not overlap. The cloud layer represents the cloud service layer, which includes high-performance computing resources connected to base stations through wired links to provide necessary resource support.

The entire system operates in discrete time with equal time slot intervals [42]. In each time slot, the mobile edge computing servers can establish communication with vehicles within its coverage area. According to literature [14], in this paper, the base station signal coverage area is sufficiently large and that the cloud server can offer services to all vehicles. For vehicles outside the coverage area of the mobile edge computing servers, tasks need to be executed on the cloud. The set of RSUs is K={1,2,…,k}, and the set of vehicles can be represented by N={1,2,…,n}. Vehicles generate computational tasks at random for each time slot. Vehicle i’s task is denoted by $T_{i}=\left(D_{i},C_{i},\Gamma_{i}\right)$. In this case, $D_{i}$ represents the task’s size, its computational complexity is denoted by $C_{i}$, and the task’s maximum tolerated delay is denoted by $\Gamma_{i}$. Table 1 gives the main symbols and their definitions.

### 3.1. Communication Model

In the vehicular edge computing system, computational services are time-divisionallly allocated to vehicles [42]. Each vehicle is given a specific time slot for data transmission as the communication period is divided into *T* time slots. Using the Shannon formula, the data transmission rate from vehilcle *i* to the connected *j* within a given time slot *t* is given by

(1) $$V_{i,j}\left(t\right)=\frac{B_{j}^{mec}}{I(t)}\cdot lb\left(1+\frac{p_{up}\cdot g_{j}}{\frac{B_{j}^{mec}}{I(t)}\cdot \sigma^{2}}\right).$$

In the aforementioned equation, several parameters are involved. $B_{i}^{mec}$ represents the total bandwidth available for the uplink channel of mobile edge computing server *j*. I(t) representes the number of tasks offloaded to mobile edge computing server during time slot *t*. While $g_{j}$ stands for the channel gain between vehicle and the mobile edge computing server *j*, $p_{up}$ refers to vehicle *i*’s transmission power. The communication channel’s noise power is represented by $\sigma^{2}$. Similarly, vehicles can also communicate with the cloud server via 5G/LTE technology. The transmission rate from vehicle *i* to the cloud server is given by

(2) $$V_{i,c}\left(t\right)=B^{cloud}\cdot lb\left(1+\frac{p_{up}\cdot g_{c}}{B^{cloud}\cdot \sigma^{2}}\right),$$

where $g_{c}$ is the channel gain between the vehicle and the cloud server, and $B^{cloud}$ denotes the bandwidth of the cloud server.

### 3.2. Computation Model

We use a partial offloading strategy in our vehicular edge computing system, where for each time slot *t*, the computational task generated by the vehicle is divisible. $\theta_{i}\left(t\right)$ is the offloading proportion of task $T_{i}$, and $\left(1-\theta_{i}\left(t\right)\right)$ is the proportion of the task to be executed at the local terminal.

The connection between vehicles and mobile edge computing servers is limited by the signal coverage range. The mobile edge computing server *j* in our vehicular edge computing system has a fixed location represented by a coordinate $q_{j}\left(t\right)={\left[q_{j}^{x}\left(t\right),q_{j}^{y}\left(t\right)\right]}^{T}\in R^{2\times 1}$, with an $r_{j}$ signal coverage radius. In time slot *t*, $p_{i}\left(t\right)={\left[p_{i}^{x}\left(t\right),p_{i}^{y}\left(t\right)\right]}^{T}\in R^{2\times 1}$ represents vehicle *i*’s location. The position of vehicle *i* after a time interval of Δt is $p_{i}\left(t+\Delta t\right)={\left[q_{i}^{x}\left(t\right)+v_{i}\left(t\right)\cdot \cos d_{i}\left(t\right)\cdot \Delta t,q_{i}^{y}\left(t\right)+v_{i}\left(t\right)\cdot \sin d_{i}\left(t\right)\cdot \Delta t\right]}^{T}\in R^{2\times 1}$, where $v_{i}\left(t\right)$ is the vehicle’s speed and $d_{i}\left(t\right)$ is the direction of movement, supposing that the vehicles in the same direction and speed during time slot *t*. For a Δt time interval in time slot *t*, only when both ${∥p_{i}\left(t\right)-q_{j}∥}^{2}$ and ${∥p_{i}\left(t+\Delta t\right)-q_{j}∥}^{2}$ are less than $r_{j}$, vehicle *i* is regarded as being inside mobile edge computing server *j*’s signal coverage. For vehicles located outside the mobile edge computing server’s signal coverage, their tasks needs to be on the cloud for execution.

#### 3.2.1. Local Computational Model

In time slot *t*, the local task execution time of task $T_{i}$ for vehicle *i* is

(3) $$T_{i}^{local}\left(t\right)=\frac{\left(1-\theta_{i}\left(t\right)\right)\cdot C_{i}\left(t\right)}{f_{local}},$$

where $C_{i}$ represents the computational complexity, and $f_{local}$ is the vehicle’s terminal execution capability. The local energy consumption is given by

(4) $$E_{i}^{local}\left(t\right)=P_{local}\cdot T_{i}^{local}\left(t\right),$$

where $P_{local}$ represents the local energy cost power of vehicle.

#### 3.2.2. Mobile Edge Computing Computational Model

We assume that the feedback delay can be disregarded due to the fact that the resulting data size is significantly smaller than the task data itself [13]. Therefore, in time slot *t*, the transmission and execution delay on the mobile edge computing server are the two parts of the total delay of task $T_{i}$. The transmission delay of task $T_{i}$ is given by

(5) $$T_{tr_{i,j}}^{mec}\left(t\right)=\frac{\theta_{i}\left(t\right)\cdot D_{i}\left(t\right)}{V_{i,j}\left(t\right)},$$

where $\theta_{i}\left(t\right)$ denotes the offloading proportion, $D_{i}\left(t\right)$ represents the task data size, and $V_{i,j}\left(t\right)$ represents the transmission rate from vehicle *i* to the connected mobile edge computing server *j*, assuming the server has *I* cores with the same processing power indicated by $f_{mec}$ for each core. When the offloaded tasks to mobile edge computing server are more than cores available at time slot *t*, the remaining tasks must be executed on the cloud. The execution delay for tasks offloaded to the mobile edge computing server is described as follows:

(6) $$T_{exc_{i,j}}^{mec}\left(t\right)=\frac{\theta_{i}\left(t\right)\cdot C_{i}\left(t\right)}{f_{mec}}.$$

When a vehicle offloads a task to the MEC, the total delay and energy consumption is depicted as

(7) $$T_{i,j}^{mec}\left(t\right)=T_{tr_{i,j}}^{mec}\left(t\right)+T_{exc}^{mec}\left(t\right),$$

(8) $$E_{i,j}^{mec}\left(t\right)=P_{tr}^{mec}\cdot T_{tr_{i,j}}^{mec}\left(t\right)+P_{exc}^{mec}\cdot T_{exc}^{mec}\left(t\right),$$

where $P_{tr}^{mec}$ and $P_{exc}^{mec}$ are the MEC’s transmission and energy consumption powers, respectively.

#### 3.2.3. Cloud Computational Model

The computational capabilities of the cloud server are specified as $f_{\mathrm{cloud}}$. For task $T_{i}$ excuted on the cloud, the execution time is marked as $T_{{\mathrm{exc}}_{i}}^{\mathrm{cloud}}\left(t\right)$, while the transmission time is represented as $T_{\mathrm{tr}i}^{\mathrm{cloud}}\left(t\right)$. The transmission and execution delay are depicted as

(9) $$T_{{tr}_{i}}^{cloud}\left(t\right)=\frac{\theta_{i}\left(t\right)\cdot D_{i}\left(t\right)}{V_{i,c}\left(t\right)},$$

(10) $$T_{exc_{i}}^{cloud}\left(t\right)=\frac{\theta_{i}\left(t\right)\cdot D_{i}\left(t\right)}{f_{cloud}}.$$

When a vehicle offloads a task to the cloud, the total delay and energy consumption are depicted as

(11) $$T_{i}^{cloud}\left(t\right)=T_{tr_{i}}^{cloud}\left(t\right)+T_{exc_{i}}^{cloud}\left(t\right),$$

(12) $$E_{i}^{cloud}\left(t\right)=P_{tr}^{cloud}\cdot T_{tr_{i}}^{cloud}\left(t\right)+P_{exc}^{cloud}\cdot T_{exc_{i}}^{cloud}\left(t\right).$$

Overall, for the task offloaded part,

(13) $$T_{i}^{offload}\left(t\right)=a_{i,j}\left(t\right)\cdot T_{i,j}^{mec}\left(t\right)+b_{i}\left(t\right)\cdot T_{i}^{cloud}\left(t\right),$$

(14) $$E_{i}^{offload}\left(t\right)=a_{i,j}\left(t\right)\cdot E_{i,j}^{mec}\left(t\right)+b_{i}\left(t\right)\cdot E_{i}^{cloud}\left(t\right),$$

where $a_{i,j}\left(t\right)$ indicates whether task $T_{i}$ is offloaded to mobile edge computing *j*, with a value of 1 if it is, otherwise 0. $b_{i}\left(t\right)$ represents whether task $T_{i}$ is offloaded to the cloud. Therefore, for task $T_{i}$,

(15) $$T_{i}\left(t\right)=\max\left\{T_{i}^{local}\left(t\right),T_{i}^{offload}\left(t\right)\right\},$$

(16) $$E_{i}\left(t\right)=E_{i}^{local}\left(t\right)+E_{i}^{offload}\left(t\right).$$

### 3.3. Problem Description

The weighted sum of the user service cost and system energy consumption is used in this paper to define the system’s overall cost. For time slot *t*, the user service cost is defined as the entire waiting time for all tasks to complete:

(17) $$W\left(t\right)=\sum_{i\in N}\sum_{j\in K}T_{i}\left(t\right).$$

The system energy consumption is defined as

(18) $$E\left(t\right)=\sum_{i\in N}\sum_{j\in K}E_{i}\left(t\right).$$

In this paper, the objective is to improve system efficiency by minimizing the system cost. According to the above model, we formulate the optimization problem:

G:minC=∑t=1Mα(t)·W(t)+β(t)·E(t)(19)      =∑t=1M∑i∈N∑j∈Kα(t)·Ti(t)+β(t)·Ei(t),s.t.(19a)C1:   maxTilocal(t),Tioffload≤Γi(t),(19b)C2   ∑j∈Kai,j(t)+bi(t)=1,(19c)C3   ∑i∈Nai,j(t)≤Ij,(19d)C4   ∑t=1M∑i∈N∑j∈KDi(t)·ai,j(t)+bi(t)=D,(19e)C5   α(t)+β(t)=1,(19f)C6   θi(t)∈[0,1],(19g)C7   α(t)∈[0,1],β(t)∈[0,1],(19h)C8   ai,j(t)∈0,1,bi(t)∈0,1,

where *C*1 is the delay constraint that means the task waiting time cannot exceed the maximum tolerant delay; *C*2 and *C*8 are the offloading constraints, which indicate the offloading destination of task $T_{i}$; *C*3 is the mobile edge computing server core constraint; *C*4 is the computational constraint, which mandates that all computation tasks be finished within the allotted time; *C*5 and *C*7 are weight coefficient constraints; and *C*6 is the offloading proportion constraint, which indicates the value range of offloading proportion.

## 4. Deep-Deterministic-Policy-Gradient-Based Task Offloading Decision-Making Algorithm

In this section, first, we suggest the vehicular edge computing system’s reinforcement learning framework, and elaborate the main elements of the Markov decision process. We also explain how to train an effective task offloading decision-making algorithm within the vehilce edge computing system. In detail, we introduce the normalization pre-processing of the states and illustrate the training and validation process of the algorithm.

### 4.1. Vehicular-Edge-Computing-Based Reinforcement Learning Framework

**Figure 2:**
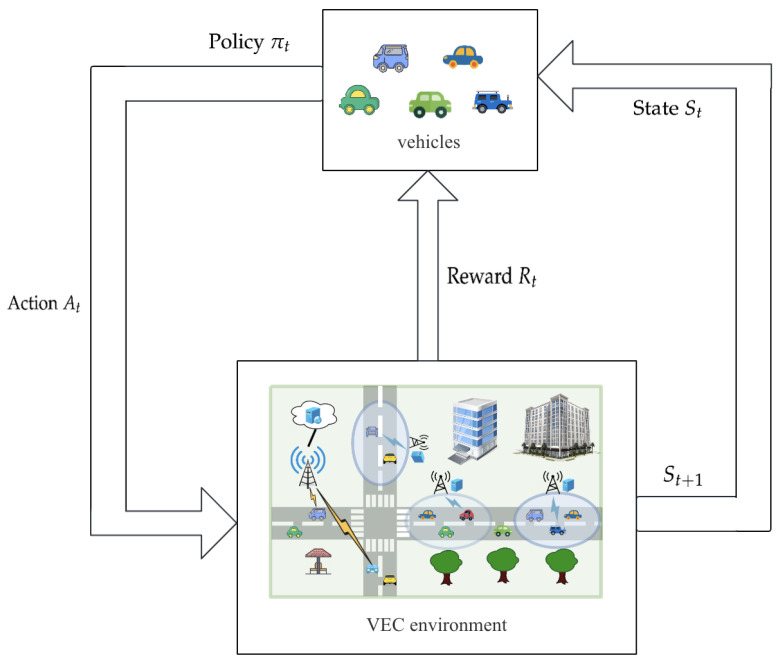
Reinforcement learning framework model based on vehilce edge computing system.

The reinforcement learning framework model of our vehicular edge computing system is shown in Figure 2. The described model represents how intelligent agent vehicles interact with environment. The agent observes the state $s_{t}$ and determines the appropriate action $a_{t}$ according to the trained policy $\pi_{t}$. After the action $a_{t}$ is chosen, the environment’s state transitions from $s_{t}$ to $s_{t+1}$. Then, the intelligent agent vehicles receive an instantaneous reward $r_{t}$ associated with the transition. This process can be abstracted and modeled as a Markov decision process, where actions and states follow the Markov property.

The basic model of the Markov decison process is a five-tuple: <S,A,P,R,γ>. In this formulation, *S* represents the system’s state space, while *A* represents the action space. $P(s_{t+1}|s_{t},a_{t})$ is the state transition function, which determines the probability of transitioning from state $s_{t}$ to $s_{t+1}$ when the agent performs action $a_{t}$. $R(s_{t},a_{t})$ determines the instantaneous reward obtained when optimal action $a_{t}$ is performed in state $s_{t}$. γ, which ranges between zero and one, indicates the extent to which the present reward $R(s_{t},a_{t})$ affects the future. The goal of the Markov decision process framework is to determine an optimal policy $\pi_{s_{t}}$ for each state $s_{t}$ that maximizes the expected cumulative long-term reward. This long-term reward is determined by the discounted sum of future rewards.

(20) $$R_{t}=\sum_{i=t}^{T}\gamma^{i-t}r\left(s_{i},a_{i}\right).$$

To assess the value of the current state in terms of long-term reward, value functions are utilized. There are two main types of value functions: the action value function $Q^{\pi }\left(s_{t},a_{t}\right)$ and the state value function $V^{\pi }\left(s_{t}\right)$.

$V^{\pi }\left(s_{t}\right)$ represents the expected cumulative reward of following policy $\pi_{s_{t}}$ starting from state $s_{t}$. $Q^{\pi }\left(s_{t},a_{t}\right)$ denotes the expected discounted return of the future of following policy $\pi_{s_{t}}$ and taking action $a_{t}$, starting from state $s_{t}$. $V^{\pi }\left(s_{t}\right)$ and $Q^{\pi }\left(s_{t},a_{t}\right)$ can also be represented by the Bellman equation, which indicates the relationship between the value of the current and the subsequent states.

(21) $$V^{\pi }\left(s_{t}\right)=E_{s_{t+1}∼E,a_{t}∼\pi }\left[r\left(s_{t},a_{t}\right)+\gamma V^{\pi }\left(s_{t+1}\right)\right],$$

(22) $$V^{\pi }\left(s_{t}\right)=E_{s_{i>t}∼E,a_{i\geq t}∼\pi }\left[R_{t}|s_{t}\right],$$

(23) $$Q^{\pi }\left(s_{t},a_{t}\right)=E_{s_{t+1}∼E}\left[r\left(s_{t},a_{t}\right)+\gamma E_{a_{t+1}∼\pi }\left[Q^{\pi }\left(s_{t+1},a_{t+1}\right)\right]\right],$$

(24) $$Q^{\pi }\left(s_{t},a_{t}\right)=E_{s_{i>t}∼E,a_{i>t}∼\pi }\left[R_{t}|s_{t},a_{t}\right].$$

The total number of potential future returns is estimated by variables $V^{\pi }\left(s_{t}\right)$ and $Q^{\pi }\left(s_{t},a_{t}\right)$. As mentioned previously, the goal of the Markov decision process framework is to discover the optimal policy, and the effectiveness of the policy is assessed using its associated value function. The optimal value function is the one that reflects the best course of action. Specifically, there are two types of optimal value functions:

(25) $$V^{*}\left(s_{t}\right)=\max_{\pi }V^{\pi }(s_{t}),$$

(26) $$Q^{*}\left(s_{t},a_{t}\right)=\max_{\pi }Q^{\pi }\left(s_{t},a_{t}\right).$$

Then, the optimal policy is

(27) $$\pi^{*}\left(s_{t}\right)=\underset{a_{t}\in A}{\arg\max}Q^{*}\left(s_{t},a_{t}\right).$$

For the optimal policy, the Bellman equation becomes the Bellman optimality equation:

(28) $$V^{*}\left(s_{t}\right)=\max_{a_{t}\in A}E_{s_{t+1}∼E,\pi }\left[r\left(s_{t},a_{t}\right)+\gamma V^{*}\left(s_{t+1}\right)\right],$$

(29) $$Q^{*}\left(s_{t},a_{t}\right)=E_{s_{t+1}∼E,\pi }\left[r\left(s_{t},a_{t}\right)+\gamma V^{*}\left(s_{t+1}\right)\right].$$

### 4.2. Markov Decision Process Elements

Based on the above model, we consider all vehicles of the vehicular edge computing system as a centralized controlled agent that can make effective offloading decisions with global information and the system environment state. The definitions of the Markov decision process elements are as follows:

(1) State space: At a time slot, the system state is
  (30) $$s_{t}=\left\{D_{remain}\left(t\right),request\left(t\right),p\left(t\right),q_{1}\left(t\right),\ldots ,q_{n}\left(t\right),D_{1}\left(t\right),\ldots ,D_{n}\left(t\right),C_{1}\left(t\right),\ldots ,C_{n}\left(t\right)\right\}.$$
  The coordinate location of the mobile edge computing server is p(t), $q_{i}\left(t\right)$ Here, $D_{remain}\left(t\right)$ represents the remaining task data size that the system needs to complete, and request(t) denotes the services that vehicles have requested. p(t) is the mobile edge computing server’s coordinate location, $q_{i}\left(t\right)$ is the vehicle *i*’s coordinate location, $D_{i}\left(t\right)$ indicates the task data size, and $C_{i}\left(t\right)$ represents the computation complexity.
(2) Action Space: A set of available actions for agents under centralized control within the given time period are represented by the action space. The actions performed by the agent can include selecting vehicles to request service, making offloading decisions, and determining offload proportion for vehicle tasks. The representation of the action is
  (31) $$a_{t}=\left\{O_{1}\left(t\right),\ldots ,O_{n}\left(t\right);\theta_{1}\left(t\right),\ldots ,\theta_{n}\left(t\right)\right\}.$$
  Here, $O_{i}\left(t\right)=1/0$ indicates whether to offload to a mobile edge computing server or a cloud server, and the task offloading proportion is represented by $\theta_{i}\left(t\right)\in \left[0,1\right]$
(3) Reward: The optimization goal of the vehicular edge computing system’s reward function is to minimize system cost while training the reinforcement learning agent to maximize long-term rewards. Consequently, according to Formula (19), the agent’s reward function is
  (32) $$r_{t}=-\sum_{i\in N}\sum_{j\in K}\alpha \left(t\right)\cdot T_{i}\left(t\right)+\beta \left(t\right)\cdot E_{i}\left(t\right).$$

### 4.3. TODM_DDPG Architecture

**Figure 3:**
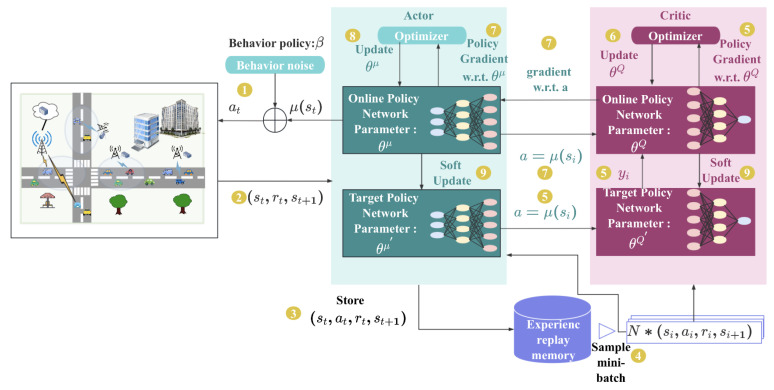
Diagram of TODM_DDPG.

In the VEC system, we utilize the actor–critic architecture-based deep reinforcement learning algorithm DDPG for our task offloading decision-making strategy. As shown in Figure 3, the algorithm iteratively trains the policy network (actor) and the Q network (critic) by interacting with the environment. By taking into account the system’s state and action space, this strategy enables the agent to learn optimal offloading decisions.

In the TODM_DDPG algorithm, the actor network consists of a target policy network in addition to an online policy network. Action $a_{t}$ that maximizes relevant action value $Q(s_{t},a_{t})$ based on state $s_{t}$ is produced by the online policy network. The actor network aims to learn actions with higher *Q* values, indicating better performance.

The critic network comprises online and target Q network. The state-action pair $\left(s_{t},a_{t}\right)$’s action value is estimated by the online Q network as $Q(s_{t},a_{t})$. The goal is to train the critic network to produce more accurate *Q* values, indicating better estimation.

To ensure training stability, the TODM_DDPG algorithm employs a delayed update technique called soft update. By combining them with the weights of the respective online networks, the target policy network and target Q network’s weights progressively update.

The TODM_DDPG algorithm’s training process can be outlined in the following way:

(1) On the basis of the behavior policy β, the actor network first chooses action $a_{t}$ and sends it to the vehicular edge computing environment for execution.
  (33) $$a_{t}=\mu \left(s_{t}|\theta^{\mu }\right)+N_{t}.$$
  Here, behavior policy β guides the environment to perform an action in the training phase. By introducing noise into the decision mechanism of action, it takes into account both exploration and exploitation to explore potentially superior policy. The online policy network generated policy $\mu \left(s_{t}|\theta^{\mu }\right)$ in the previous stage, while $\theta^{\mu }$ is the parameter. $N_{t}$ is Gaussian noise with mean $n_{0}$ and variance $\sigma_{0}^{2}$.
(2) The agent of vehicular edge computing environment executes action $a_{t}$ and returns instantaneous reward $r_{t}$ and new environment state $s_{t+1}$.
(3) To train the online network, the actor network records the state transition process $\left(s_{t},a_{t},r_{t},s_{t+1}\right)$ as the dataset.
(4) N transition data $\left(s_{i},a_{i},r_{i},s_{i+1}\right)$ is randomly sampled as mini-batch training data from replay memory buffer R for the online network.
(5) Based on actions $\mu^{{}^{\prime }}\left(s_{i+1}\right)$ generated by the target policy network and the transition data, the target Q network first calculates label value $y_{i}$.
  (34) $$y_{i}=r_{i}+\gamma Q^{\prime }\left(s_{i+1},\mu^{\prime }\left(s_{i+1}|\theta^{\mu^{\prime }}\right)|\theta^{Q^{\prime }}\right).$$
  Gradient ${▿}_{\theta^{Q}}L$ is then calculated by the online Q network using the back-propagation method in the neural network and loss function $L(\theta^{Q})$:
  (35) $$L\left(\theta^{Q}\right)=E_{\mu^{\prime }}\left[{\left(y_{i}-Q\left(s_{i},a_{i}|\theta^{Q}\right)\right)}^{2}\right].$$
(6) Online Q network’s parameters $\theta^{Q}$ are updated.
(7) Firstly, according to action $a=\mu \left(s_{i}\right)$, the online Q network computes gradient ${\left.\nabla_{a}Q\left(s,a|\theta^{Q}\right)\right|}_{s=s_{i},a=\mu \left(s_{i}\right)}$. Then, the online policy network calculates the policy gradient:
  (36) $${\left.{\left.\nabla_{\theta^{\mu }}J\approx \frac{1}{N}\sum_{i}\nabla_{a}Q\left(s,a|\theta^{Q}\right)\right|}_{s=s_{i},a=\mu \left(s_{i}\right)}\nabla_{\theta^{\mu }}\mu \left(s|\theta^{\mu }\right)\right|}_{s_{i}}.$$
(8) The online policy network’s parameters $\theta^{\mu }$ are updated.
(9) Using the sliding mean approach, the target policy and Q network parameters $\theta^{\mu }$ and $\theta^{Q}$ are softly updated:
  (37) $$\theta^{Q^{\prime }}\leftarrow \tau \theta^{Q}+\left(1-\tau \right)\theta^{Q^{\prime }},$$
  (38) $$\theta^{\mu^{\prime }}\leftarrow \tau \theta^{\mu }+\left(1-\tau \right)\theta^{\mu^{\prime }}.$$
  Here, $\tau \in \left[0,1\right]$.

### 4.4. State Normalization

Based on the aforementioned TODM_DDPG algorithm’s training process, two different DNN networks are utilized to to fit the value and policy function of the actor and critic networks, respectively, as illustrated in Figure 3. Taking the actor network as an example, we can observe that it takes $s_{t}$ as input and outputs action $a_{t}=\mu \left(s_{t}\right)$. However, the distribution of activation input values of the DNN network changes during the training process, gradually approaching the top and lower boundaries of the range of nonlinear function values [43]. This leads to the disappearance of gradients in the lower layers during the back-propagation process, resulting in slow convergence. To better train the neural network, in addition to using the Relu activation function, based on batch normalization [44,45], Algorithm 1 is proposed to normalize input states $s_{t}$ with different ranges.

**Algorithm 1:** State Normalization
 **Input**:
 State parameters: $D_{remain}\left(t\right)$, p(t), $q_{i}\left(t\right)$, $D_{i}\left(t\right)$, $C_{i}\left(t\right)$;
 Scale factors: $\lambda_{1}$, $\lambda_{2}$;
 Min–Max value: $Min_{D}$, $Min_{C}$, $Max_{D}$, $Max_{C}$;
 **Output**:
 Normalized State parameters: ${\hat{D}}_{remain}\left(t\right)$, $\hat{p}\left(t\right)$, ${\hat{q}}_{i}\left(t\right)$, ${\hat{D}}_{i}\left(t\right)$, ${\hat{C}}_{i}\left(t\right)$;
${\hat{D}}_{remain}\left(t\right)$←$D_{remain}\left(t\right)$/$\lambda_{1}$;$\hat{p}\left(t\right)\leftarrow p\left(t\right)/\lambda_{2}$,   ${\hat{p}}_{i}\left(t\right)\leftarrow q_{i}\left(t\right)\;/\lambda_{2}$;${\hat{D}}_{i}\left(t\right)\leftarrow \left(D_{i}\left(t\right)-Min_{D}\right)/\left(Max_{D}-Min_{D}\right)$;${\hat{C}}_{i}\left(t\right)\leftarrow \left(C_{i}\left(t\right)-Min_{C}\right)/\left(Max_{C}-Min_{C}\right)$;**Return** ${\hat{D}}_{remain}\left(t\right)$,$\hat{p}\left(t\right)$,${\hat{q}}_{i}\left(t\right)$,${\hat{D}}_{i}\left(t\right)$,${\hat{C}}_{i}\left(t\right)$;


This paper normalizes the state parameters with different ranges separately. $\lambda_{1}$ and $\lambda_{2}$ are scaling factors used to normalize the remaining total data size $D_{remain}\left(t\right)$ and coordinate information, respectively. The task data size and complexity are normalized using the min–max normalization method. The top and lower boundaries of the task data volume and complexity for each time slot are denoted by variables $Min_{D}$, $Min_{C}$, $Max_{D}$, and $Max_{C}$, respectively.

### 4.5. TODM_DDPG Training and Validating Algorithm

We propose the training algorithm, which is depicted in Algorithm 2, based on the aforementioned TODM_DDPG algorithm and state normalization method. By iteratively adjusting the online policy network parameters $\theta^{\mu }$ and online Q network parameters $\theta^{Q}$ through interactions with the environment, actor, and critic networks throughout the training phase, the algorithm seeks to maximize the long-term reward and returns the optimal online policy network parameter $\theta^{\mu }$ after training. Algorithm 3 describes the validation algorithm for computing the offloading strategy, which utilizes trained optimal parameters $\theta^{\mu }$. It performs the offloading decision-making process and obtains the system cost based on the output policy of the training algorithm.
**Algorithm 2:** TODM_DDPG training algorithm
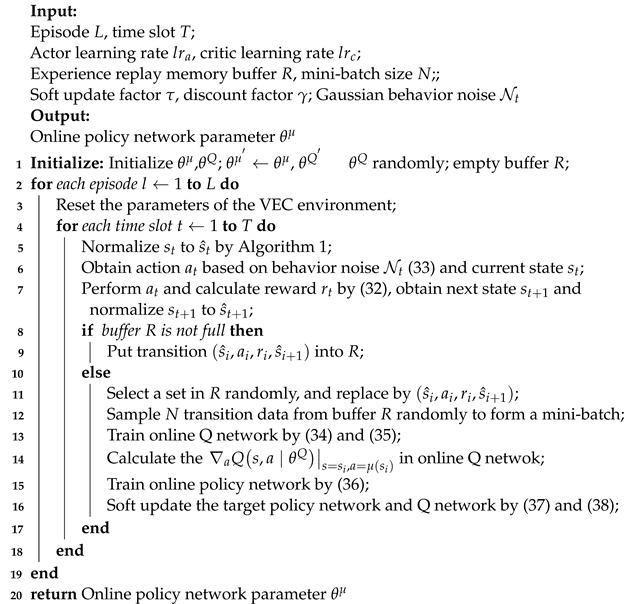


**Algorithm 3:** TODM_DDPG validating algorithm

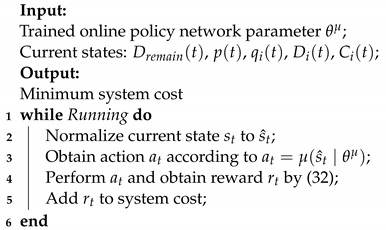



## 5. Experimental Analysis

### 5.1. Parameter Setting

We evaluate the algorithm in the Python development environment based on the Tensorflow platform in order to assess the efficiency and operational performance of the algorithm model in this research. In the vehicular edge computing test system, the simulation scenario is a three-tier structure of cloud-edge-vehicle consisting of one cloud server, one edge server and several vehicle users. Among them, a two-dimensional square area covered by the base station signals is considered, and within the area, the mobile edge computing servers are distributed on the road side, and their signal coverage is a circular area. Within the base station’s coverage area, vehicles move at random along the road. We use TensorFlow to initialize an instance of the deep reinforcement learning agent function acccording to the settings of Markov decision elements in Section 4.2. Table 2 displays the environmental parameters as well as the neural network’s parameters.

### 5.2. Result and Analysis

#### 5.2.1. Parameter Analysis

Since the selection of different hyperparameters impacts the effectiveness, convergence, and stability of the algorithm, the significance of hyperparameter setting should be considered. In order to obtain the ideal values for the hyperparameters used in the TODM_DDPG algorithm, we conducted a series of experiments.

**Figure 4:**
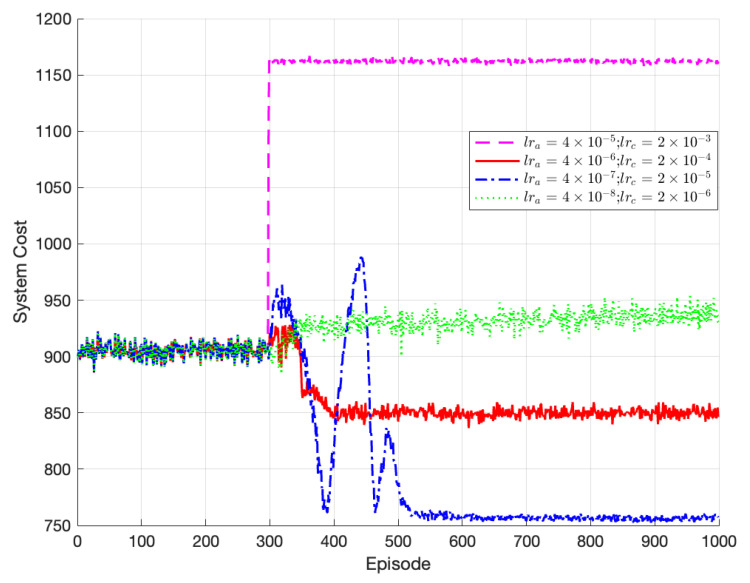
Performance of TODM_DDPG algorithm under different learning rates.

Figure 4 demonstrates the impact of different learning rates on the convergence and stability of the algorithm. Since the actor network is updated by the critic network, we assumed that the learning rate of the critic network is greater than that of the actor network. Two combinations of learning rates were compared: $lr_{a}=4\times 10^{-7}$ and $lr_{c}=2\times 10^{-5}$, and $lr_{a}=4\times 10^{-6}$ and $lr_{c}=2\times 10^{-4}$. The algorithm converges in both cases, but the solution obtained with $lr_{a}=4\times 10^{-7}$ and $lr_{c}=2\times 10^{-5}$ is superior to the one with $lr_{a}=4\times 10^{-6}$ and $lr_{c}=2\times 10^{-4}$. The latter converges to a local optimum instead of the global optimum. This is because the larger update rates for both the critic and actor networks allow for larger update steps, enabling convergence to the local optimum. Alternatively, using smaller learning rates like $lr_{a}=4\times 10^{-8}$ and $lr_{c}=2\times 10^{-9}$ maintains stability but leads to poor optimization performance. The lower learning rate slows down the network’s update speed, requiring more iterations to converge. Consequently, it fails to reach the optimal solution. Based on the experiment, the optimal network learning rate selected was $lr_{a}=4\times 10^{-7}$ and $lr_{c}=2\times 10^{-5}$.

**Figure 5:**
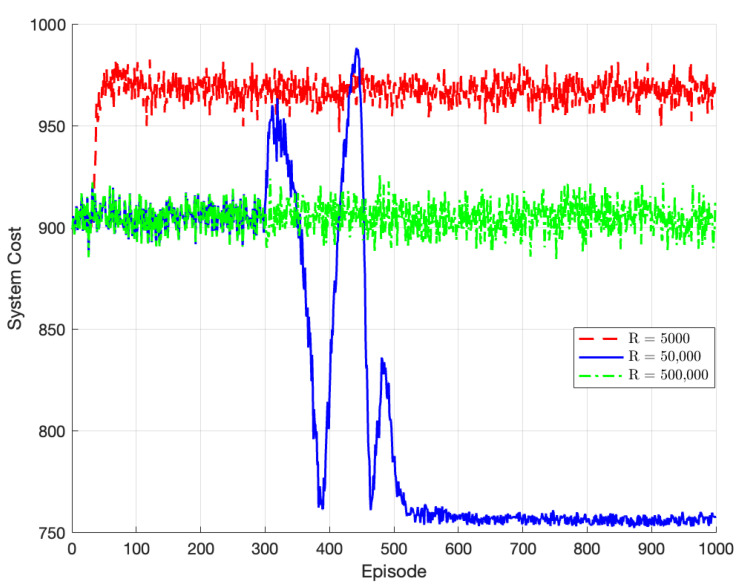
Performance of TODM_DDPG algorithm under different experience replay buffer sizes.

Figure 5 compares the algorithm’s performance for experience replay buffers of different sizes, denoted by R. When R = 5000, the algorithm reaches a local optimum around episode 70. However, due to the small size of the experience replay buffer, it fails to extract sufficient data feature information, leading to an inability to learn the optimal strategy. When R = 50,000, the algorithm converges to the global optimum at around episode 520. When R = 500,000, the algorithm fails to converge due to the excessively large experience replay buffer, resulting in a longer training time and insufficient data updates. Therefore, the optimal size of the experience replay buffer chosen in this paper is 50,000.

**Figure 6:**
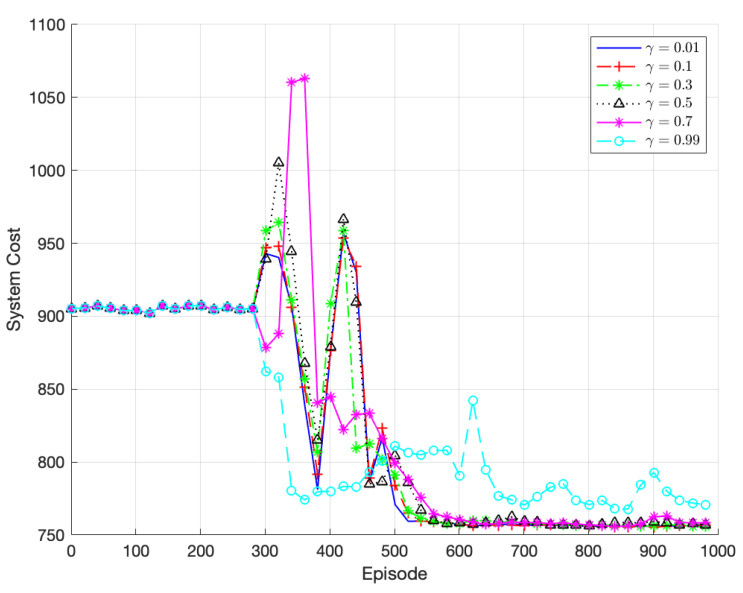
Performance of TODM_DDPG algorithm under different discount factors.

Figure 6 presents the comparison of the algorithm’s performance for various discount factors γ. The algorithm achieves the fastest convergence and best performance when γ = 0.01. This is attributed to the dynamic nature of the vehicular edge computing system, where environmental conditions change over time. Considering long-term returns over the entire duration does not accurately represent long-term behavior, resulting in significant variations in data across different time periods and an inability to capture comprehensive data features. Therefore, this paper adopts γ = 0.01 as the optimal discount factor.

**Figure 7:**
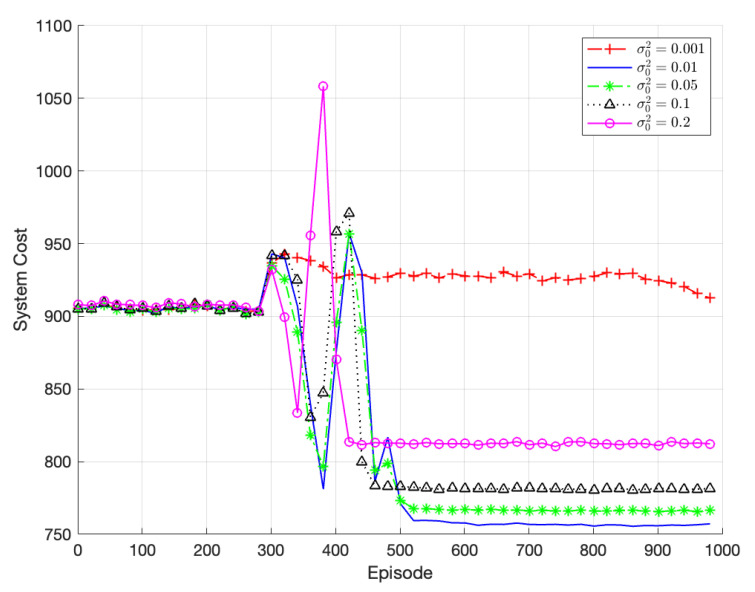
Performance of TODM_DDPG algorithm under different exploration parameters.

Figure 7 illustrates the comparison of the algorithm’s performance for different exploration parameters $\sigma_{0}^{2}$, which represents the exploration noise in the TODM_DDPG algorithm. Since the policy network outputs deterministic actions, exploration in TODM_DDPG relies on adding noise to the action space. In this case, the noise is represented by the variance parameter $\sigma_{0}^{2}$. When $\sigma_{0}^{2}$ is set to 0.01, the algorithm eventually converges to the best result. Notably, as $\sigma_{0}^{2}$ increases, the algorithm explores a larger action space, leading to faster convergence but a potentially larger noise distribution space. As shown in the figure, when $\sigma_{0}^{2}$ is set to 0.2, the algorithm exhibits fluctuations around a system cost of approximately 820. When $\sigma_{0}^{2}$ is set to 0.001, the exploration parameter is too small, resulting in a narrow range of generated action space, which can lead to convergence to local optima or an inability to converge. Therefore, the optimal exploration parameter chosen in this paper is $\sigma_{0}^{2}$ = 0.01.

**Figure 8:**
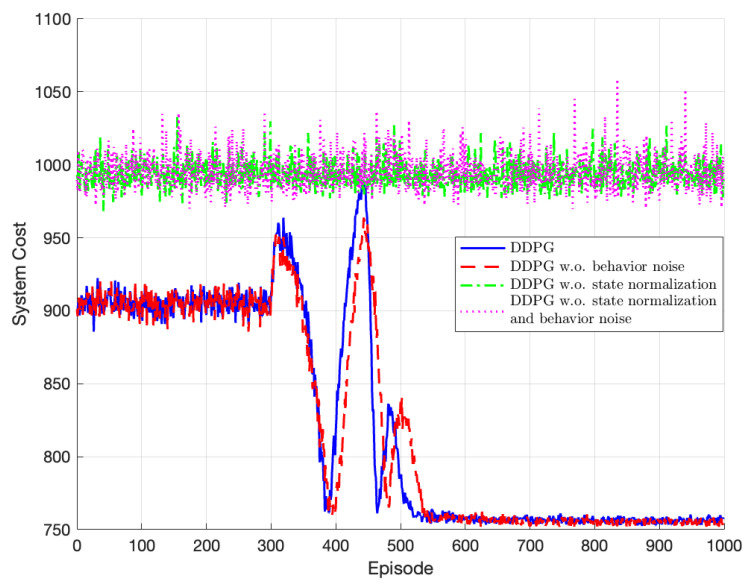
Performance of TODM_DDPG algorithm with state normalization or behavior noise.

Figure 8 presents the comparison of the algorithm’s performance with the influence of state normalization and behavior noise during training. As we mentioned in Section 4.3, the DDPG training process executed by introducing noise into the decision-making mechanism of actions considers both exploration and exploitation to explore potentially superior policies. In the figure, the algorithm’s convergence speed slows down when behavior noise is not used during policy training. Furthermore, the presence of state normalization has a more significant effect than that of behavioral noise. As mentioned before, TODM_DPPG utilizes two different deep neural network networks to fit the value and policy function of actor and critic networks, respectively. Taking the actor network as an example, we can observed that it takes the state as input and outputs policy actions. However, the distribution of the activation input values of the deep neural network changes during the training process, gradually approaching the upper and lower bounds of the non-linear function value range. This causes gradients in lower layers to vanish during the backpropagation process, leading to slow convergence. Therefore, for the characteristics of the deep neural network, we propose Algorithm 1, which incorporates normalization of the state values using scaling factors and the min–max method. Training the algorithm without state normalization, large state parameters can cause slow convergence of the deep neural network, rendering the training process ineffective, and the algorithm approximate to be greedy.

#### 5.2.2. Performance Comparison

**Figure 9:**
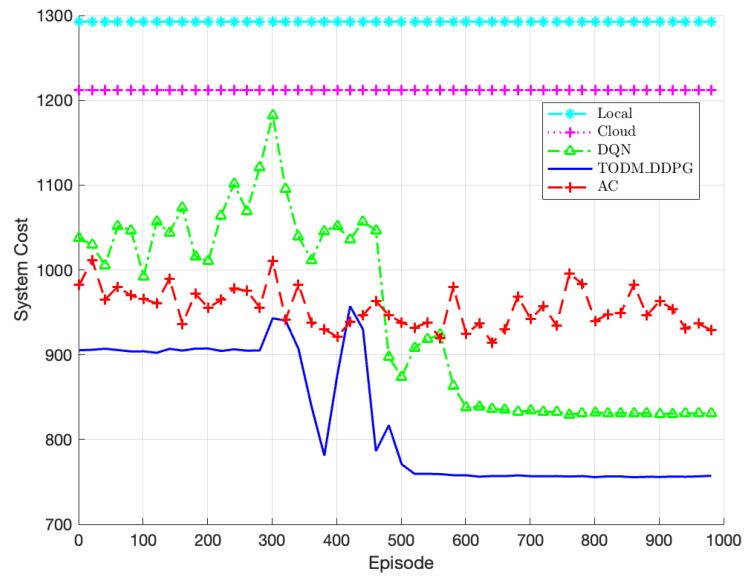
Performance of system cost of different algorithm under different episodes.

The comparison of performance of different algorithms is shown in Figure 9. We compare five different algorithms in this experiment: the AC algorithm, the TODM_DDPG algorithm, the DQN algorithm, the local offloading algorithm, and the cloud offloading algorithm. For the three reinforcement learning algorithms, the training iterations are set to 1000. It is evident from the figure that the three reinforcement learning algorithms implement significantly lower system costs compared to the local offloading and cloud offloading algorithms. However, the AC algorithm fails to converge during the training process due to the interaction between the actor and critic. This is because the action of the actor relies on the critic’s value, and updating both networks simultaneously when the critic’s value is challenging to converge may leads to instability. On the other hand, both the TODM_DDPG and DQN algorithms, with their dual network structures, effectively overcome this issue by breaking the correlation among training data and achieving convergence. However, the DQN algorithm fails to converge to the minimum system cost. This is mainly due to the fact that the DQN algorithm discretizes the action space of continuous actions, resulting in an inability to find the optimal offloading strategy accurately. In comparison, the TODM_DDPG algorithm performs best. Its ability to explore continuous action spaces enables it to find optimal solutions more effectively. Therefore, the TODM_DDPG algorithm demonstrates superior performance in terms of minimizing system cost.

**Figure 10:**
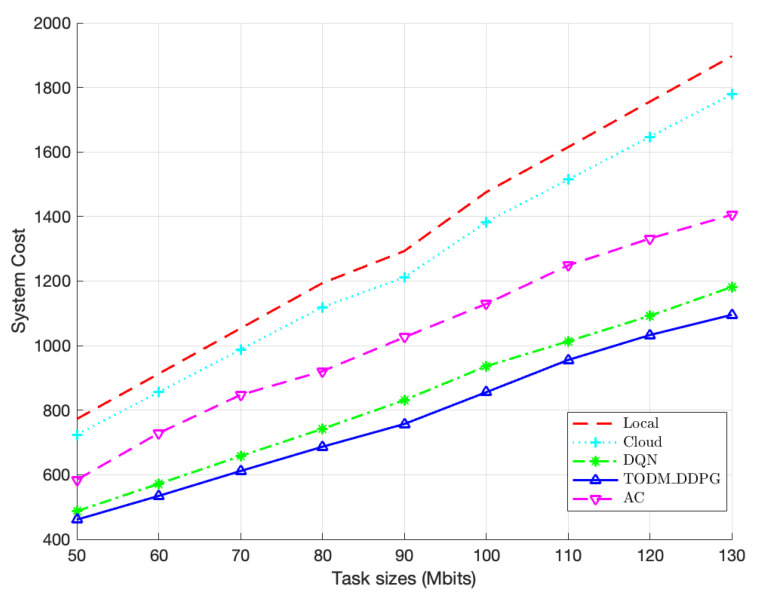
Performance of system cost of different algorithm under different different task size ranges.

Figure 10 illustrates the comparison of performence of the algorithms under different task size ranges. The system costs of all algorithms increase as the task size increases, showing a positive correlation. Within a given task data size range, the TODM_DDPG algorithm’s system cost is significantly lower compared to those of local or cloud offloading algorithms, as these offloading algorithms fail to fully utilize system resources. Furthermore, from the figure, we can observe that the TODM_DDPG algorithm’s advantage over other methods in terms of system cost becomes more obvious as the task data size increases. Overall, the TODM_DDPG algorithm consistently converges to the minimum system cost.

**Figure 11:**
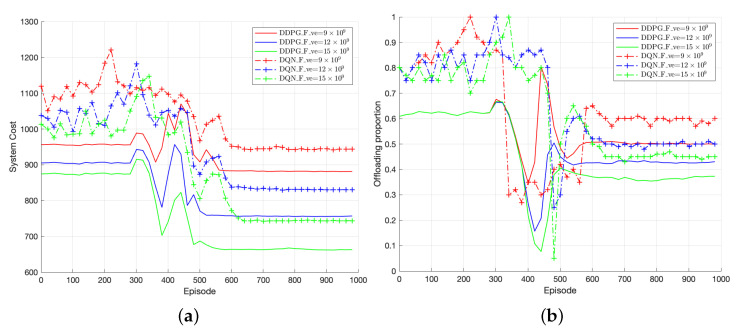
(**a**) Performance of system cost of TODM_DDPG and DQN under different vehicle computing capacity. (**b**) Performance of offloading proportion of TODM_DDPG and DQN.

Figure 11 depicts the performance comparison of the proposed solution (DQN and TODM_DDPG) in terms of system cost and offloading proportion under different vehicle computing capacities. Figure 11a shows the convergence performance comparison between TODM_DDPG and DQN approaches under different vehicle computing capacities. The AC algorithm is not compared because of its lack of convergence. It can be observed that when the vehicle’s computing capacity is relatively low, i.e., $F_{ve}$ = 9 × 10${}^{9}$, both optimization approaches result in higher system costs compared to when the vehicle’s computing capacity is $F_{ve}$ = 12 × 10${}^{9}$ and $F_{ve}$ = 15 × 10${}^{9}$. On the other hand, as shown in Figure 11b, when the vehicle’s computing capacity is larger, the offloading proportion in the system is smaller. Therefore, as the vehicle’s computing capacity increases, the vehicles tend to execute tasks locally. Smaller computing capacities result in slower data processing speeds in the system at a given time, leading to larger maximum delays between local execution and offloading, i.e., higher offloading rates.

Additionally, since DQN algorithms are suitable for discrete action spaces, the offloading proportion level of DQN is set to {0,0.05,0.1,…,1.0} in this paper. Based on Figure 11a, we can find that the TODM_DDPG scheme achieves lower system cost compared with the DQN scheme under different vehicle computing capabilities. Also, in Figure 11b, we can see that the DQN algorithm can only output a limited discrete action set for offloading proportion, while TODM_DDPG is capable of continuous action output. This is because TODM_DDPG outputs the value of the action by adding a layer of policy network on the basis of the DQN algorithm instead of directly outputting the maximum Q value like DQN, and thus expands to the control space of continuous actions. Hence, for the same vehicle computing capacity, the TODM_DDPG algorithm exhibits lower system costs compared to the DQN algorithm, as shown in Figure 11a.

**Figure 12:**
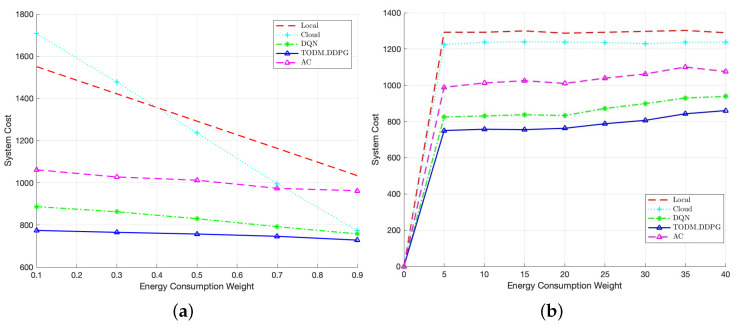
(**a**) Performance of system cost of different algorithms under different energy consumption weights. (**b**) Performance of system cost of different algorithms under different number of vehicles.

Figure 12a represents the performance comparison of different algorithms under different energy consumption weights. The figure shows that the TODM_DDPG algorithm always achieves the lowest system cost, regardless of the weight coefficients. Additionally, we can find that with the increase in the energy weight coefficient, the system cost of the cloud offloading algorithm decreases significantly, and the system costs of DQN and TODM_DDPG algorithms gradually approach that of the cloud offloading algorithm. The reason for this is that when the energy weight is larger, the advantage of offloading tasks to the cloud in terms of system cost becomes more significant compared to local offloading. Therefore, the DQN and TODM_DDPG algorithms tend to offload tasks to the cloud or teh edge for executing. As for the AC algorithm, due to its lack of convergence, the optimization effect is not significant as the energy consumption weight increases. Figure 12b demonstrates the performance comparison of different algorithms under different numbers of vehicles. In this case, we assume that in regard to the MEC processing capacity, MEC cores *I* are equal to seven, and under different numbers of vehicles, the total task size to be completed in whole time period is the same. In the figure, we can find that as the number of vehicles increases, and when the number of vehicles is less than 25, the average system cost of all schemes is almost constant, and when the number of vehicles is greater than 25, the system costs of the three reinforcement learning algorithm schemes begin to increase. This is because, as the number of vehicles increases, the amount of offloaded tasks also increases, and the MEC cores are limited, which leads to redundant tasks being offloaded to the cloud server for processing and increases the system cost. In addition, it can be concluded from the figure that the system cost of the proposed TODM_DDPG algorithm is less than those of the other four algorithms, because the TODM_DDPG algorithm can find the optimal value in the continuous action space and obtain the optimal offloading proportion.

## 6. Conclusions

In this paper, the computation offloading problem of vehicular edge computing environment is studied, and a vehicular edge computing system based on three-layer cloud edge vehicle architecture is proposed. Based on the deep deterministic policy gradient, this paper suggests a task offloading decision-making algorithm, namely TODM_DDPG. We provide a comprehensive description of the algorithm’s training and testing processes, effectively addressing the challenge of high-dimensional continuous action space. Furthermore, we introduce a state normalization method to enhance the algorithm’s convergence and stability. Subsequently, we conduct experiments to examine the effects of several fundamental hyperparameters on the algorithm’s performance, and the results are contrasted with those obtained from baseline algorithms. The testing results prove the effectiveness of our suggested approach in reducing system costs. The algorithm evaluation considers the joint optimization of delay and energy, and points out the effectiveness of the strategy under different task sizes and vehicle computing capabilities. The TODM_DDPG algorithm is superior to the other four schemes and obtains lower system cost and more accurate task offloading rate, which shows its effectiveness in optimizing task offloading for vehicular edge computing. Moreover, for a scalable and long-term vehicular edge computing system, the proposed scheme shows high reliability even under different energy consumption and delay weights and the number of vehicles.

In future research, we plan to explore additional aspects such as task types that consider dependencies and mobile edge computing server offloading request forwarding and channel resource allocation, aiming to simulate real-world scenarios more accurately. For this dependency-aware task offloading, the problem of task offloading is transformed into two sub-problems of offloading decision and resource allocation. Firstly, the offloading positions of subtasks are determined according to the dependencies between subtasks. Secondly, the resource allocation optimization process is formulated as a Markov decision process. Based on this, our goal is to design a hybrid computational offloading strategy for resource allocation and task allocation using a collaborative mechanism, taking into account both task offloading and resource allocation, so that sub-tasks can be executed in parallel and the optimal fine-grained offload strategy can be obtained.

## Figures and Tables

**Table 1:** The Meaning of the Main Symbols.

Symbol	Interpretation
*K*	The set of RSUs, K={1,2,…,k}
*N*	The set of vehicles, N={1,2,…,n}
$T_{i}$	Vehicle i’s task, $T_{i}=\left(D_{i},C_{i},\Gamma_{i}\right)$, i∈N
$D_{i}$	Data size of task $T_{i}$
$C_{i}$	Computational complexity of task $T_{i}$
$\Gamma_{i}$	Maximum tolerated delay of task $T_{i}$
*V*	Data transmission rate
*B*	Bandwidth
*I*	The number of tasks offloaded to mobile edge computing server
$p_{up}$	Vehicle i’s transmission power
*g*	Channel gain
$\sigma^{2}$	The communication channel’s noise power
$\theta_{i}$	The offloading proportion of task $T_{i}$
$q_{j}$	The location of mobile edge computing server *j*, $q_{j}={\left[q_{j}^{x},q_{j}^{y}\right]}^{T}$
$p_{i}$	The location of vehicle *i*, $p_{i}={\left[p_{i}^{x},p_{i}^{y}\right]}^{T}$
*f*	Execution capability
*P*	Energy cost power
$a_{i,j}$	The flag of task $T_{i}$ offloaded to mobile edge computing server *j*
$b_{i}$	The flag of task $T_{i}$ offloaded to the cloud

**Table 2:** Experimental parameters.

Parameter	Values	Parameter	Values
Task generation probabilities	0.3	Vehicle num *N*	10
BS coverage	1200 × 1200 m${}^{2}$	RSU coverage radius	500 m
RSU coordinate	[500, 500]	Vehicle speed *v*	36 km/h
$B^{mec}$	90 MHz	$B^{cloud}$	60 MHz
MEC cores *I*	3	Noise power $\sigma^{2}$	−100 dBm
MEC channel gain $g_{m}$	−52 db	Cloud channel gain $g_{c}$	−54 db
$p_{up}$	0.2 W	$P_{tr}^{mec}$/$P_{tr}^{cloud}$	0.3 W
$P_{local}$	0.6 W	$P_{exc}^{mec}$/$P_{exc}^{cloud}$	0.5 W
*D*	[90, 100] Mbits	*C*	[9, 10] Gcycles
$f_{local}$	1.2 GHz	$f_{mec}$	2.5 GHz
$f_{cloud}$	5 GHz	Delay weight α	0.5
Energy weight β	0.5	$\lambda_{1}$	$1.61\times 10^{10}$
$\lambda_{2}$	1200	Layers	3
Neurons	400, 300, 10	Learning rate $lr_{a}$	$4\times 10^{-7}$
Learning rate $lr_{c}$	$2\times 10^{-5}$	Discount factor γ	0.01
Batch size	64	Replay memory buffer R	$5\times 10^{5}$
Exploration parameter $\sigma_{0}^{2}$	0.01	Soft update factor τ	0.01

## Data Availability

Not applicable.
